# Text Message Responsivity in a 2-Way Short Message Service Pilot Intervention With Adolescent and Young Adult Survivors of Cancer

**DOI:** 10.2196/12547

**Published:** 2019-04-18

**Authors:** Alexandra M Psihogios, Yimei Li, Eliana Butler, Jessica Hamilton, Lauren C Daniel, Lamia P Barakat, Christopher P Bonafide, Lisa A Schwartz

**Affiliations:** 1 The Children's Hospital of Philadelphia Philadelphia, PA United States; 2 The Children's Hospital of Philadelphia, University of Pennsylvania Philadelphia, PA United States; 3 University of Pittsburgh Pittsburgh, PA United States; 4 Rutgers University Camden, The Children's Hospital of Philadelphia Camden, NJ United States

**Keywords:** mHealth, adolescents, young adults, cancer, chronic illness, self-management

## Abstract

B**ackground:** Text message interventions hold promise for adolescents and young adults (AYAs) with chronic health conditions, including childhood cancer survivors; however, engagement is often suboptimal. Limited research has studied mobile health intervention outcomes beyond efficacy. Understanding responsivity to different types of text messages (ie, when a participant texts back) can provide practical, actionable information to optimize engagement in future projects.

**Objective:**

Within a 2-way text messaging study in AYAs who recently completed treatment for cancer, we sought to evaluate text message responsivity across different types of text messages.

**Methods:**

AYAs who recently completed treatment for cancer (n=26; mean age=16 years; 62% female, 16/26 participants) received 2-way text messages about survivorship health topics over a 16-week period. Using participants’ text message log data, we coded responsivity to text messages and evaluated trends in responsivity to unprompted text messages and prompted text messages of varying content (eg, medication reminders, appointment reminders, and texts about personal experiences as a cancer survivor).

**Results:**

Across prompted and unprompted text messages, responsivity rapidly decreased (*P* ≤.001 and =.01, respectively) and plateaued by the third week of the intervention. However, participants were more responsive to prompted text messages (mean responsivity=46% by week 16) than unprompted messages (mean responsivity=10% by week 16). They also demonstrated stable responsivity to certain prompted content: medication reminders, appointment reminders, goal motivation, goal progress, and patient experience texts.

**Conclusions:**

Our methodology of evaluating text message responsivity revealed important patterns of engagement in a 2-way text message intervention for AYA cancer survivors.

## Introduction

### Background

Adolescents and young adults (AYAs) send and receive text messages more often than any other age demographic [1,2]. They also represent a cohort with an elevated risk for a number of maladaptive health behaviors, including poor disease self-management and nonadherence among those with chronic health conditions [3]. Mobile health (mHealth) interventions involving text messaging are particularly appealing to AYAs [4-6] and offer practical and scalable solutions for improving their health behaviors within real-world environments [7-9]. Indeed, text messaging interventions demonstrate modest but significant improvements in health knowledge and behaviors across AYAs with a variety of chronic health conditions [7,10]. Unfortunately, despite AYAs’ enthusiasm about text messaging and the proliferation of text message interventions, their objective engagement with text messaging interventions is often low [11,12]. There is no simple formula for designing interactive and engaging text messages, and continuous user engagement or *stickiness* represents a pervasive challenge [13,14]. To promote sustained AYA engagement in text message interventions, further research is needed to determine whether patterns of text message responsivity vary across prompted, unprompted, and content-specific (such as medication reminders) text messages. Using a 2-way short message service (SMS) intervention with AYA survivors of childhood cancer, we capitalized on an opportunity to evaluate trends in responsivity to different types of text messages over a 16-week period.

To date, limited mHealth research has moved beyond efficacy evaluations to rigorously examine user engagement [15], such as text message responsivity. We define text message responsivity as instances in which a participant sends a response text after receiving a prompted (requests a response) or unprompted (does not explicitly request a response) text message from the research team. Outside of traditional health care settings, AYA engagement with text messages must contend with other aspects of their daily life and their fluctuating motivation to engage in purposeful health behaviors [15]. Many texting interventions require repeated exposure to health information and reminders to promote health behavior change. However, AYAs may habituate to this information and experience competing demands from other frequently used mobile apps, leading to disengagement with the intervention [16-18]. Further investigation into AYAs’ responsivity to different types of text messages is critical, as the strength and durability of mHealth intervention effects are intricately connected to participant engagement [19-22].

Currently, the mHealth literature offers limited methodological guidance on how to analyze and optimize user engagement [15], including responsivity to text messages. Little attention has been paid to dismantling and evaluating text message components, such as the content that is most likely to elicit a text back from participants, which would deepen our understanding of how text messages function to improve health and well-being [15]. As such, to our knowledge, this paper represents the first to operationalize the term text message responsivity. One text message intervention for adolescents with type 1 diabetes (*Sweet Talk*) evaluated unprompted messages that were sent from participants to the research team [11]. In this study, participants were more likely to send certain types of unprompted text messages (eg, submission of blood glucose values) compared with others (eg, questions about their diabetes). These authors concluded that participants valued 2-way text message capabilities but were most motivated to submit disease monitoring information. A more fine-grained evaluation of responsivity to different types of prompted and unprompted text messages will help to further delineate the content that may engage (or reengage) the end user in an intervention over time.

AYA cancer survivors represent an exemplar group for SMS interventions. AYA survivors must continue lifelong follow-up care, establish preventative health behaviors, and adhere to medication regimens to manage a host of secondary morbidities of their curative cancer treatment (ie, late effects) [23,24]. At the same time, they are often transient during young adulthood and live great distances from their treating hospital, leaving them with difficulties with access to appropriate follow-up care [25]. As such, digital health interventions (eg, mHealth, Web-based, and social media) have been evaluated as methods of overcoming traditional barriers to AYA survivors’ engagement in follow-up care and to promote healthy behaviors such as exercise [26,27]. There is a growing body of literature supporting the feasibility, acceptability, and initial efficacy of mHealth interventions in this population, including 1 study of a text message system that delivered survivorship information and resources [28] and a separate study of an app-based symptom management intervention [29]. Yet, engaging AYA cancer survivors can be challenging [30], and research has yet to thoroughly investigate their engagement in mHealth interventions [26].

### Objectives

This paper addresses a literature gap by assessing AYA childhood cancer survivors’ responsivity to specific text message content over the course of a 16-week 2-way pilot SMS intervention called *T*exting *H*ealth *R*esources to *I*nform, moti*V*ate, and *E*ngage (THRIVE). Consistent with past text messaging interventions, we expected a significant trend for decreasing responsivity over the course of the intervention. However, given that tailored text message interventions yield larger effect sizes than generic ones [31,32], we hypothesized that AYAs would demonstrate greater responsivity to prompted text messages that were tailored. By evaluating AYA responsivity to different types of text messages, we contribute to the literature by (1) illustrating a potentially generalizable method of analyzing objective text message engagement data and (2) providing guidance about maximizing the engagement of AYA childhood cancer survivors in an SMS intervention.

## Methods

### Intervention Development

THRIVE text messages were designed to support theory-informed categories of inform (eg, information about health promotion, late effects, and resources), motivate (eg, providing encouragement and monitoring of health goal attainment), and engage (eg, engagement in follow-up care, sharing experiences, autonomy promotion, and psychosocial support). THRIVE was grounded in the Social-ecological Model of AYA Readiness to Transition to Adult Care (SMART) [33]. SMART is a validated model of AYA self-management that emphasizes multilevel influences on AYA self-management and transition to adult-centered care, including knowledge, self-efficacy, supportive relationships, and goals. The health belief model [34] and social cognitive theory [35] also influenced THRIVE text message development. Consistent with these health behavior theories, text messages were intended to enhance awareness of health vulnerability and importance of continued engagement in follow-up care and to motivate and reinforce positive health behaviors.

Text messages were developed by a research team comprising clinical psychologists, nurse practitioners, oncologists, and student (young adult age) research assistants and volunteers. The content, limited to 160 characters and written at a sixth-grade reading level, was similar to health-related information in the hospital-based AYA Survivor Handbook provided to controls. All team members reviewed text messages individually and in weekly team meetings for a period of approximately 2 months to ensure clarity of messages and consistency with influential theories and study aims. The pilot text messages were then sent to all team members for a trial period to test the system and further review the content.

This process resulted in the creation of 210 text messages that included content on healthy eating, exercise, sleep, sun safety, risk-taking behavior (drugs, alcohol, and sexual activity), academic and social life after cancer treatment, engagement in follow-up medical care, and connecting with other survivors and content relevant to 1 of the following health goals selected by the participant: (1) healthy eating, (2) smoking cessation, (3) reengage in school, (4) reengage in social activities, (5) increase physical activity, and (6) improve sleep/fatigue. Text messages were tailored by age or goal (23% of messages) and interactive (41%) in that they prompted the participant to text back a response to receive additional information, answer a survey item, or answer a trivia question; the remaining messages were unprompted and/or generic. Participants with ongoing medication regimens and upcoming clinic appointments also received weekly medication adherence texts and appointment reminder texts. Participants could also spontaneously send an unprompted text to the study team. A separate manuscript of our proof-of-concept randomized controlled trial describes intervention development in more detail (Schwartz, LA, unpublished data, February 2019). Participants who were randomized to THRIVE reported high acceptability.

### Participants

This manuscript analyzed data from the THRIVE intervention group only. The inclusion criteria were as follows: (1) must be within 1 year of completing cancer treatment; (2) must be in cancer remission; (3) must be aged between 12 and 25 years; (4) must be able to read and speak English; and (5) for AYAs aged under 18 years, must have a parent/caregiver provide consent for participation. Of the 31 patients in the intervention group, 26 patients received text messages and were included in this analysis. Exclusion reasons included never turning on the phone/received text messages (n=3), relapsed on study (n=1), and determined history of nonmalignant, genetic tumor diagnosis (n=1).

### Procedure

After obtaining institutional review board approval, eligible participants were identified using the cancer center’s patient registry and upcoming clinic appointment schedules. Before recruitment, the patients’ primary oncology providers were contacted to confirm eligibility. Participants were approached and invited to participate during outpatient oncology visits. After participants and caregivers (when applicable) provided informed consent and assent, participants were asked to select a health-related goal to pursue over the course of the study (eg, increase physical activity, improve healthy eating, and improve sleep/fatigue). Participants randomized to the intervention group were provided with an iPhone (an older version to reduce cost) and received 1 to 2 daily text messages over the course of 16 weeks. At the time of study initiation, our institution requested that we provide participants with a secured iPhone to ensure privacy and protect personal data (eg, cell phone numbers) from a third-party vendor. The frequency and duration of the text messages were informed by similar SMS interventions for AYAs with other chronic diseases [32,36,37]. Participants were able to select the time of the day to receive text messages and additional appointment reminders. Text messages were automatically sent by a tailored text message platform designed by an outside vendor (Reify Health), who was contracted to provide technical infrastructure and support. A research assistant monitored the delivery of the texts and incoming texts. Log data of 2-way text communication were securely stored and downloaded from the Reify Health platform.

**Table 1:** Text message examples by category.

Text message category		Example message
**Prompted messages**		
	Trivia^a^	Does smoking help you lose weight? Text back 1 for yes or 2 for no.
	More information^a^	Losing your hair during treatment is hard for most patients with cancer. If you lost hair, text back 1 for info on your hair after cancer.
	Patient experience^a^	Your treatment & some medications can make skin more sensitive to sun than it used to be. Did you apply sunscreen today? Text back 1 if yes, or 2 if no.
	Goal-tailored	Think about your physical activity level now compared to 3 months ago - text back if you feel 1 better, 2 the same, or 3 worse
	Age-tailored	Managing the healthcare system & learning about insurance can be tricky. Text 1 to learn more about health insurance as a young adult & survivor.
	Goal motivation^a^	How motivated are you to be more active? Text back: 1 - Not at all, 2 - Slightly, 3 - Somewhat, 4 - Very, 5 - Extremely
	Goal progress^a^	How much progress have you made on your goal to be more active? Text back: 1-None, 2-small amount, 3-moderate amount, 4-a lot, 5-huge amount
	Medication reminder^a^	Have you been taking your medication? Text 0 - I am not taking medication anymore, 1 - No, not really, 2 - sometimes, 3 - always.
	Appointment reminder^a^	You have an appointment to come to CHOP in 2 days, on 11/28/2014 at 3:00PM. Do you know how you are getting there? Text back 1 for Yes or 2 for No.
**Unprompted messages**		
	Information	Feeling tired right after treatment is totally normal. Healing takes energy & time. Make sure to listen to your body & pace yourself throughout the day.
	Goal-tailored	Put physical activity in your schedule - setting aside a specific time of the day will help prevent you from putting it off until later.
	Age-tailored	Feeling connected when you go back to school can be tough. Join a club or sport that interests you to find peers who enjoy similar things.

^a^Analyzed responsivity to this type of prompted text message.

### Data Analytic Plan

We exported SMS raw log data for each participant in Microsoft Excel format, which delineated the date, time, and content of every text message the participant received from the research team and every message the participant sent to the research team. Moreover, 2 trained study staff coded responsivity to text messages by week, that is, how many times each participant responded to a text message by text message category. After coding the text message responsivity, for each participant at each week, we calculated the overall percentage responsivity (ie, a continuous variable of the total number of responses to text messages divided by the total number of texts received). Similarly, for each participant at each week, we calculated percentage responsivity to prompted (ie, messages that requested a response) and unprompted (ie, messages that did not request a response) text messages. We further analyzed select prompted text messages about (1) medication reminders (as applicable), (2) appointment reminders (as applicable), (3) goal motivation, (4) goal progress, (5) health knowledge *trivia*, (6) patient experiences, and (7) requests for *more information* (see Table 1). We did not analyze goal-tailored or age-tailored texts as the prompted text messages in these categories were accounted for in patient experience and more information categories (ie, these categories were not mutually exclusive).

For text message responsivity outcomes measured weekly (overall, prompted, unprompted, and more information text messages), we constructed a longitudinal piecewise linear regression model, with the participant percentage responsivity at each week as the outcome. The piecewise model assumed 1 slope from weeks 1 to 3 and another slope after week 3. We specified this piecewise model based on descriptive data and graphs about responsivity over time, which suggest a rapid decrease in responsivity in weeks 1 to 3 and attenuated decrease afterward. Testing the significance of each slope suggests whether there was a significant decreasing or increasing trend of text message responsivity in each of the 2 periods. We also tested the significance of difference in the slopes for after versus before week 3 to evaluate if a reduced model of the longitudinal linear model should be used. The generalized estimation equation method with exchangeable correlation structure was used to account for the potential within-subject correlations among the repeated outcomes over weeks. For the other responsivity outcomes with less frequent measures, longitudinal linear regression models were constructed to test the significance of the slope in terms of whether there was a significant decreasing or increasing trend of text message responsivity over time. All analyses were performed in SAS 9.4.

## Results

### Demographic Information

Means, SDs, ranges, and percentages for demographic and medical information are presented in Table 2. The average age of participants was 16 years and the majority had completed cancer treatment for a leukemia or lymphoma diagnosis (14/26 participants, 54%). On average, participants had completed cancer therapy 5 months before their enrollment in the study.

### Overall Responsivity

**Figure 1:**
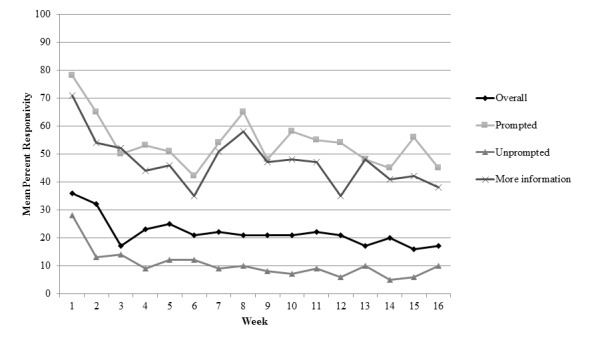
Average responsivity across text messages categories received weekly over a 16-week period.

Means and SDs of percentage responsivity by week for each type of text message (ie, overall, prompted, unprompted, and prompted content) are provided in Table 3. When evaluating text message responsivity across all types of prompted and unprompted text messages, there was a rapid decrease in responsivity in the first 3 weeks of the intervention, with a 7% decrease per week (95% CI −10.7 to −3.0; *P*<.001), and a much attenuated decrease after week 3, with a 0.4% decrease per week (95% CI −0.7 to −0.1; *P*=.003; Figure 1). The slopes before and after week 3 were significantly different (*P*=.002), supporting the use of the piecewise linear model over the linear model. Descriptively, at week 1, the mean responsivity across all text messages was 36% (SD 22.4%), and by week 16, the mean responsivity decreased to 17% (SD 17.9%; see Table 3).

### Responsivity to Prompted and Unprompted Messages

In terms of responsivity to prompted text messages, there was again a significant and rapid decrease in responsivity in the first 3 weeks of the intervention, with a 13% decrease per week (95% CI −19.5 to −6.3; *P*<.001), which plateaued after week 3 (decrease 0.1% per week; 95% CI −0.8 to 0.6; *P*=.79; see Figure 1). The slopes before and after week 3 were significantly different (*P*<.001). Participants demonstrated relatively high engagement with prompted text messages at week 1 but with significant variability (mean responsivity=78%, SD 31%). By week 16, the average responsivity to prompted text messages decreased to 46% (SD 37%; see Table 3).

Similarly, there was a rapid decrease in responsivity to unprompted text messages in the first 3 weeks, with a 7% decrease per week (95% CI −11.9 to −1.5; *P*=.01), and an attenuated decrease after week 3, with 0.4% decrease per week (95% CI −0.6 to −0.1; *P*=.01; see Figure 1). The slopes before and after week 3 were significantly different (*P*=.02). The mean responsivity at week 1 was 28% (SD 29.3%), which decreased to 10% by week 16 (SD 26.3%; see Table 3).

**Table 2:** Demographic and disease information (N=26).

Variable		Statistics, n (%)	Mean (SD)	Minimum-maximum
Current age (years)		26 (100)	16.42 (2.87)	12.00-20.00
Age at cancer diagnosis (years)		26 (100)	15.48 (3.01)	8.68-19.05
Time off treatment (months)		26 (100)	5.18 (3.58)	0.39-11.96
Gender (female)		16 (62)	—^a^	—
**Race**				
	White	17 (65)	—	—
	African American	6 (23)	—	—
	Other	2 (8)	—	—
	Asian	1 (4)	—	—
Ethnicity, Hispanic		3 (12)	—	—
**First cancer type**				
	Leukemia/lymphoma	14 (54)	—	—
	Solid tumor	7 (27)	—	—
	Brain tumor	5 (19)	—	—
Had relapse		2 (8)	—	—
Had second cancer		1 (4)	—	—

^a^For categorical variables, mean, SD, and minimum-maximum have not been listed.

**Table 3:** Means and SDs for text message responsivity (%) across categories of text messages received weekly during the intervention.

Week	Total, n; mean (SD)	Unprompted, n; mean (SD)	Prompted, n; mean (SD)	More information, n; mean (SD)
Week 1	26; 36% (22%)	25; 28% (29%)	26; 78% (31%)	24; 71% (44%)
Week 2	26; 32% (18%)	26; 13% (20%)	26; 65% (36%)	25; 54% (43%)
Week 3	26; 17% (23%)	26; 14% (25%)	26; 50% (51%)	25; 52% (51%)
Week 4	26; 23% (18%)	26; 9% (20%)	26; 53% (36%)	26; 44% (43%)
Week 5	26; 25% (23%)	26; 12% (26%)	26; 51% (42%)	26; 46% (45%)
Week 6	26; 21% (23%)	26; 12% (26%)	26; 42% (40%)	26; 35% (42%)
Week 7	26; 22% (23%)	26; 9% (23%)	26; 54% (47%)	26; 51% (43%)
Week 8	26; 21% (24%)	26; 10% (27%)	26; 65% (49%)	26; 58% (50%)
Week 9	26; 21% (20%)	26; 8% (22%)	26; 48% (40%)	26; 47% (39%)
Week 10	26; 21% (18%)	26; 7% (17%)	26; 58% (40%)	25; 48% (49%)
Week 11	26; 22% (20%)	26; 9% (20%)	26; 55% (42%)	26; 47% (48%)
Week 12	26; 21% (18%)	26; 6% (18%)	26; 54% (32%)	26; 35% (49%)
Week 13	26; 17% (20%)	26; 10% (27%)	25; 48% (46%)	24; 48% (45%)
Week 14	26; 20% (18%)	25; 5% (13%)	26; 45% (39%)	26; 41% (40%)
Week 15	25; 16% (18%)	25; 6% (19%)	25; 56% (49%)	12; 42% (51%)
Week 16	25; 17% (18%)	25; 10% (26%)	25; 46% (37%)	25; 38% (39%)

**Table 4:** Means and SDs for text message responsivity (%) across categories of text messages received intermittently during the intervention.

Week	Medication reminders, n; mean (SD)	Appointment reminders, n; mean (SD)	Goal motivation, n; mean (SD)	Goal progress, n; mean (SD)	Patient experience, n; mean (SD)	Trivia, n; mean (SD)^a^
Week 1	17; 78% (44%)	—^b^	—	—	—	26; 89% (33%)
Week 2	—	—	26; 73% (45%)	—	25; 80% (41%)	—
Week 3	—	—	—	—	—	—
Week 4	17; 67% (50%)	2; 50% (71%)	—	26; 69% (47%)	—	—
Week 5	—	2; 50% (71%)	—	—	26; 65% (49%)	—
Week 6	—	1; 0% (0%)	26; 62% (50%)	—	1; 100% (0%)	—
Week 7	—	2; 50% (71%)	—	—	8; 50% (53%)	—
Week 8	17; 78% (44%)	1; 100% (0%)	—	26; 62% (50%)	—	—
Week 9	—	—	—	—	—	—
Week 10	—	1; 0% (0%)	26; 65% (49%)	—	9; 78% (44%)	—
Week 11	—	5; 60% (55%)	—	—	—	26; 62% (50%)
Week 12	17; 67% (50%)	6; 58% (49%)	—	26; 54% (51%)	12; 92% (29%)	—
Week 13	—	5; 70% (45%)	—	—	1; 0% (0%)	—
Week 14	—	4; 50% (58%)	25; 60% (50%)	—	1; 100% (0%)	—
Week 15	—	4; 50% (58%)	—	—	—	25; 52% (51%)
Week 16	17; 67% (50%)	5; 50% (50%)	—	14; 57% (51%)	—	—

^a^Significant linear decrease across weeks.

^b^Not applicable.

### Responsivity to Promoted Content-Specific Messages

We also examined trends in responsivity across certain types of prompted text messages (see Table 1). Participant responsivity to medication reminders (*P*=.64), appointment reminders (*P*=.31), goal motivation (*P*=.12), goal progress (*P*=.25), and patient experience texts (*P*=.74) did not significantly change over the course of the 16-week intervention, suggesting relatively stable participant engagement with these types of text messages (see Table 4). In contrast, there was a significant decrease in responsivity to *more information* texts in the first 3 weeks, with a 10% decrease per week (95% CI −17.2 to −1.8; *P*=.02), which plateaued after week 3 (decrease 0.5% per week; 95% CI −1.4 to 0.5; *P*=.33; see Figure 1). The slopes before and after week 3 were significantly different (*P*=.03). Descriptively, the average responsivity to *more information* texts at week 1 was 71%, which decreased to 38% by week 16 (see Table 3). Participants also responded to fewer health trivia texts each week, with a 3% decrease per week (95% CI −4.24 to −0.97; *Z*=−3.13; *P*=.002). Mean responsivity for trivia texts was 89% at week 1, which decreased to 52% by the last week of the intervention.

## Discussion

### Principal Findings

Over the course of a 16-week 2-way SMS intervention for AYA cancer survivors called THRIVE, we found that responsivity to text messages (overall, prompted, unprompted, and *more information* texts) peaked during the first week and rapidly decreased by week 3. However, participants were more responsive to prompted than to unprompted text messages and demonstrated stable responsivity to certain prompted content: medication reminders, appointment reminders, goal motivation, goal progress, and patient experience texts. Our analysis of text message responsivity represents an important contribution to the literature, as the majority of existing mHealth interventions have focused exclusively on efficacy data and neglected objective engagement data [38]. Our approach, involving coding how many times a participant sent a *text back* to different types of text messages, was practical, feasible to do with a small research team, and unveiled important patterns in AYA survivors’ text message responsivity.

The decline in text message responsivity is consistent with past research that has demonstrated that engagement with mHealth tools is generally low and rapidly decreases in the first few weeks of use [11,12]. For example, in a mHealth intervention called HeartSteps that delivered contextually tailored activity suggestions to sedentary adults, the effect of activity suggestions diminished over time and largely disappeared after 1 month [39]. Similarly, 74% of consumers report discontinuing the use of commercial health apps after the tenth use [40]. This study adds to this body of literature and contributes novel information about how text message responsivity declined and plateaued in an AYA cancer survivor sample, likely because of habituation. At the same time, across weeks, participants demonstrated the highest percentage responsivity to prompted text messages. Among the prompted text message content categories, participants demonstrated relatively stable engagement (generally >50%) with certain content: medication reminders, appointment reminders, goal motivation, goal progress, and patient experience texts.

The data generated by this study have furthered our understanding of AYA survivors’ objective engagement with specific text message content in a few ways. First, deteriorating responsivity in the first 3 weeks of the intervention highlights the importance of planning for habituation in future trials. To sustain long-term engagement with text messages, researchers should consider the implementation of a text message delivery schedule that minimizes the risk of habituation by favoring times when participants are most likely to be receptive and available to respond [39]. Although each participant in our study selected the timing of their text messages, employing reinforcement learning algorithms [41] would help to further personalize the timing of text message delivery based on a participant’s prior responses (eg, their responsivity to messages at certain times, days of the week, and based on certain content). Notably, sending 1 to 2 health-related text messages per day for 16 weeks may have not been ideal for establishing and sustaining the level of responsivity needed to support meaningful behavior change. Indeed, based on learning theories, it may have been beneficial to temporarily suspend text messages when a participant’s responsivity decreased (eg, at week 3 of our intervention) to reduce burden and encourage a spontaneous recovery in engagement once text messages were reinitiated [42]. Similarly, it may have been useful to vary text messages by sending engaging (noninterventional) content on some days, such as memes, gifs, and life insights that have been used to increase engagement in daily mobile assessments in other AYA populations [43].

Second, although greater responsivity to prompted text messages is somewhat intuitive as these messages requested a response, relatively few 2-way text message interventions (as opposed to 1-way text messages) have been tested to address AYA health behaviors [10] and even fewer for AYA cancer survivors [26]. Our research provides evidence that bidirectional messaging may be an important component for promoting AYA survivors’ engagement with text message interventions.

Third, AYA survivors demonstrated sustained responsivity to prompted texts about their temporal health behaviors (eg, medication adherence), a personally selected health goal, and personal experiences compared with text messages that were education based (ie, seeking more information or responding to health trivia). Such findings are consistent with evidence that tailored, personally relevant text messages are more engaging that generic text messages [11,27]. Although it is well documented that educational interventions alone are insufficient to improve health behaviors such as medical adherence [44,45], our findings show that purely informational content may have also been less engaging for AYAs in our research study. Alternatively, it is possible that participants were more engaged with these content-specific text messages because they were received less often, appeared more novel, and thus were less prone to habituation. Experimental designs such as microrandomized trials will help to disentangle how the frequency, timing, and content of tailored text messages impact AYA survivors’ proximal engagement in mHealth interventions [46,47].

### Limitations

With limited methodological guidance about how to best evaluate text message user data, we recognize that our approach represents only one of the several possible methods of examining text message responsivity. This approach is not without limitations. Our texting platform was not equipped to measure whether participants read the text messages. As a result, we relied exclusively on a text back from participants to determine their engagement, which neglects the possibility that participants passively engaged with content (especially with unprompted messages that did not request a response). To our knowledge, no research has examined differences in intervention efficacy for participants who actively respond to text messages compared with those who passively read, but did not respond, to text messages. Such research would be beneficial for illustrating dose-response relationships within a texting intervention or the amount of responsivity that is minimally needed to experience certain intervention effects [38,48]. In addition, all text messages were delivered via a secondary study iPhone, which may have resulted in lower responsivity than if messages were delivered to a personal phone.

Another notable limitation was the large variability across participants in their responsivity to text messages, as evidenced by large SDs in most text message categories. Although our focus was assessing responsivity to text messages at the group level, heterogeneity between participants highlights the importance of examining within-subject responsivity in future research. Furthermore, our investigation was underpowered to test whether specific participant characteristics (such as age, sex, or cancer diagnosis) predicted trends in text message responsivity, which could provide further insight into responsivity patterns. More research, with larger samples, is clearly needed to demonstrate whether our method of examining text message responsivity contributes meaningful knowledge about user engagement and explanatory data about the efficacy of the intervention. Despite these limitations, this generalizable approach unveiled practical information about how to enhance our text bank for AYA cancer survivors before a scaled-up randomized controlled trial (ie, increase the number of prompted text messages and expand tailoring of text messages to increase personal relevance).

### Conclusions and Future Directions

This study illustrates 1 method for analyzing user engagement with 2-way text messages and contributes knowledge about the types of text messages that AYA survivors responded to the most. The analysis of participant responsivity to text messages, as well as other mHealth log data (eg, such as opening a mobile app, clicking a link, and reading a text message), can help provide critical insight into participant engagement with mHealth technology [15]. Consistent with prior research [11,28,39], our findings showed that AYA survivors’ responsivity to text messages rapidly decreased during the first few weeks of the intervention, but they demonstrated higher engagement with prompted and personally relevant text messages. Future mHealth interventions should integrate bidirectional and tailored content to maximize AYA survivors’ engagement in text message interventions. For example, following this pilot intervention, we expanded THRIVE text messages to include educational content that was tailored to each AYA survivor’s cancer treatment history and related risks for late effects, for example:

[Name], Ever notice that you have trouble hearing the TV or other people at large gatherings? If so, let a member of your medical team know and ask to get your hearing checked. Cisplatin (a chemotherapy you received) can affect your hearing.

The second wave of this intervention is described in a separate publication [49] and is currently being tested in a randomized controlled trial.

Other intervention features may have increased responsivity, such as delivering text messages directly to an AYA’s personal phone and adding gamification elements (eg, earning points for responding to messages) [43]. Future research can systematically test the causal effects of various message delivery schedules, as well as different engagement strategies (eg, gamification and incentives), on increasing an individual’s responsivity to text messages over time [43,50]. In addition to analyzing responsivity to specific text message content, analysis of additional text message components, such as who sent the text message, when was it delivered, and how frequently were text messages sent, will help elucidate salient user patterns and preferences [15]. We recommend that future research make use of text message log data when possible and examine both intervention (eg, length of intervention, types of text messages, gamification, and target health behavior) and contextual factors (eg, participant age, sex, race/ethnicity, and health status) that influence engagement [51,52]. Future research should also evaluate the latency of responsivity, such as the elapsed time to respond to text messages, as studying response times has yielded valuable information in other health areas (eg, factors that influence health providers’ response times to patient physiological monitors) [53]. Building and sharing knowledge in this area can encourage additional research on engagement with mHealth text messages; inform important modifications to text banks; and answer multiple calls for rigorous methodological research in the development, implementation, and evaluation of user-centered mHealth tools [12,54,55].

## Abbreviations

AYAs: adolescents and young adults
mHealth: mobile health
SMART: Social-ecological Model of AYA Readiness to Transition to Adult Care
SMS: short message service
THRIVE: Texting Health Resources to Inform, motiVate, and Engage
